# Cardiac preservation using *ex vivo* organ perfusion: new therapies for the treatment of heart failure by harnessing the power of growth factors using BMP mimetics like THR-184

**DOI:** 10.3389/fcvm.2025.1535778

**Published:** 2025-03-18

**Authors:** William D. Carlson, Dattatreyamurty Bosukonda, Peter C. Keck, Philippe Bey, Shannon N. Tessier, Frederic R. Carlson

**Affiliations:** ^1^ Division of Cardiology, Mass General Hospital/Harvard, Boston, MA, United States; ^2^ Department of Medicine, Harvard Medical School, Boston, MA, United States; ^3^ Therapeutics by Design, Weston, MA, United States; ^4^ Center for Engineering in Medicine and Surgery, Massachusetts General Hospital, Harvard Medical School, and Shriners Children’s Hospital, Boston, MA, United States

**Keywords:** Bone morphogenetic protein, BMP, mimetics, THR-123, THR-184, NMP, TGF, Ex-vivo heart machine perfusion

## Abstract

As heart transplantation continues to be the gold standard therapy for end-stage heart failure, the imbalance between the supply of hearts, and the demand for them, continues to get worse. In the US alone, with less than 4,000 hearts suitable for transplant and over 100,000 potential recipients, this therapy is only available to a very few. The use of hearts Donated after Circulatory Death (DCD) and Donation after Brain Death (DBD) using *ex vivo* machine perfusion (EVMP) is a promising approach that has already increased the availability of suitable organs for heart transplantation. EVMP offers the promise of enabling the expansion of the overall number of heart transplants and lower rates of early graft dysfunction. These are realized through (1) safe extension of the time between procurement and transplantation and (2) *ex vivo* assessment of preserved hearts. Notably, *ex vivo* perfusion has facilitated the donation of DCD hearts and improved the success of transplantation. Nevertheless, DCD hearts suffer from serious preharvest ischemia/reperfusion injury (IRI). Despite these developments, only 40% of hearts offered for transplantation can be utilized. These devices do offer an opportunity to evaluate donor hearts for transplantation, resuscitate organs previously deemed unsuitable for transplantation, and provide a platform for the development of novel therapeutics to limit cardiac injury. Bone Morphogenetic Protein (BMP) signaling is a new target which holds the potential for ameliorating myocardial IRI. Recent studies have demonstrated that BMP signaling has a significant role in blocking the deleterious effects of injury to the heart. We have designed novel small peptide BMP mimetics that act via activin receptor-like kinase (ALK3), a type I BMP receptor. They are capable of (1) inhibiting inflammation and apoptosis, (2) blocking/reversing the epithelial-mesenchymal transition (EMT) and fibrosis, and (3) promoting tissue regeneration. In this review, we explore the promise that novel therapeutics, including these BMP mimetics, offer for the protection of hearts against myocardial injury during *ex vivo* transportation for cardiac transplantation. This protection represents a significant advance and a promising *ex vivo* therapeutic approach to expanding the donor pool by increasing the number of transplantable hearts.

## 1 Introduction

Heart Disease is the leading cause of death in the USA (National Center for Health Statistics 2023, https://www.cdc.gov/nchs/fastats/leading-causes-of-death.htm). In the USA in 2022, there were 702,880 deaths from heart disease and the cost of heart failure accounted for the major sub-proportion of this dread disease, with an annual incidence of congestive heart failure in the US of over 300,000 and an annual mortality of over 100,000 (1). In 2018, there were 6.2 million US adults that had heart failure and in 2020 the cost of heart failure/disease to US society was estimated to be $252.2 billion (https://www.cdc.gov/heart-disease/data-research/facts-stats/index.html).

Congestive heart failure can be caused by several different insults including acute myocardial infarction and other myocardial injuries. The annual adjusted US incidence of hospitalization for acute myocardial infarction is approximately 550,000 for first episodes (2) and 200,000 for recurrent episodes (3). Ultimately, these insults lead to contractile dysfunction mediated at a cellular level by processes including apoptosis, inflammation, and fibrosis (4). It is a complicated pathophysiology involving many different molecular messengers, affecting various cell types and includes cellular differentiation and proliferation (5).

The only definitive therapy available is a heart transplant. While this procedure is lifesaving, it has many complications and is limited by a finite supply of donor hearts. In the US, between 2000 and 2010, the number of heart transplant remained relatively constant at between 3,500 and 4,000 (6). In 2022, there were 3,789 hearts transplanted. Of the 10,000 hearts in the donor pool, approximately 6,000 were deemed unsuitable for transplantation. Because of this attrition in the donated heart pool, many patients die awaiting a suitable donor heart (6). In 2019, 20 patients on the waiting list died each day (7). If we could prevent injury and resuscitate or regenerate some of the hearts deemed unsuitable, we could decrease the time on the waiting list, increase the number of transplants, and save many more lives. In this paper we examine *ex vivo* therapeutic interventions and conventional interventions that can prevent injury and promote recovery.

## 2 Problem—ischemic injury of the *ex vivo* heart

### 2.1 Procurement and transport of hearts for transplantation

**Figure 1:**
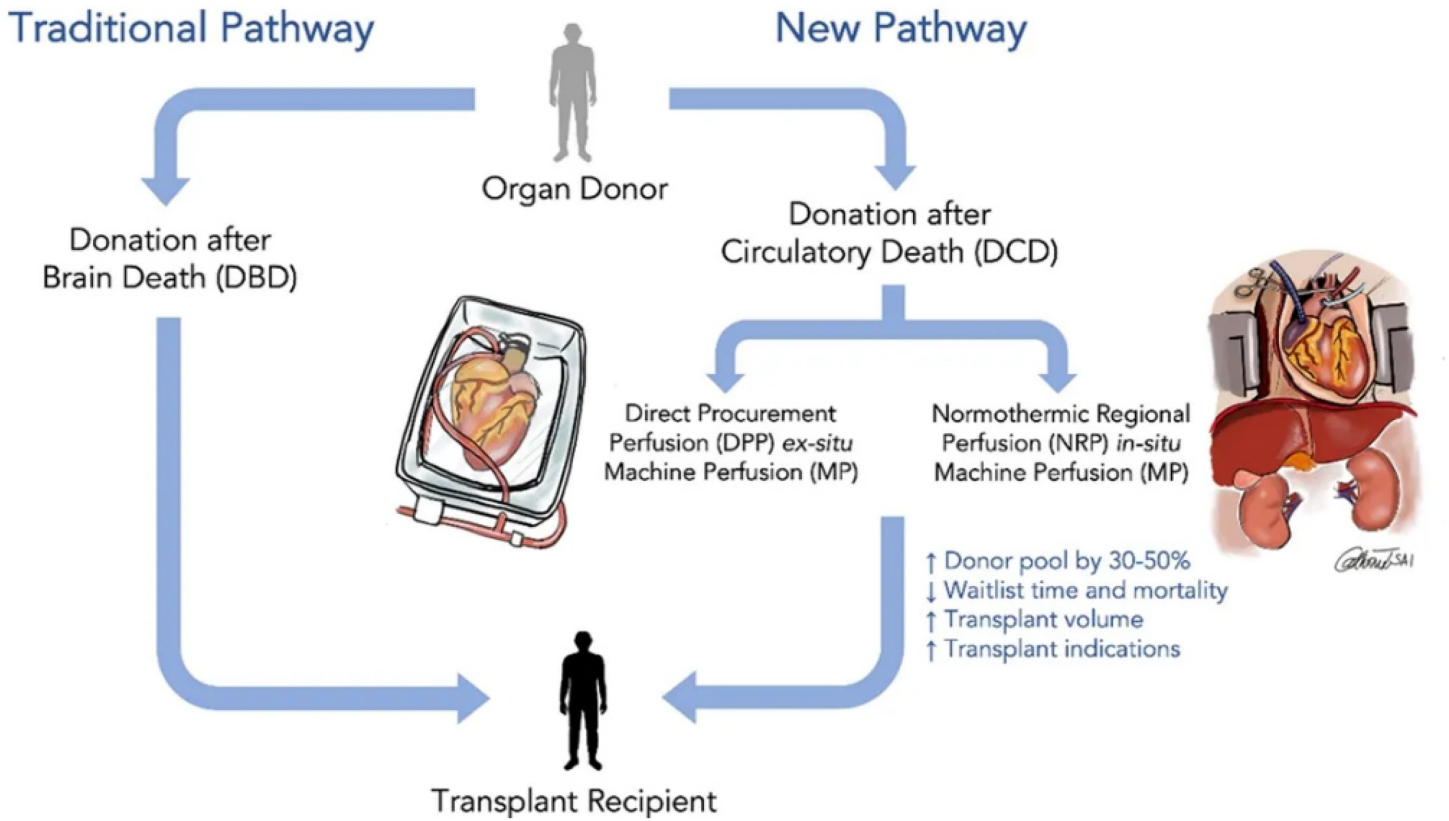
Revolutionizing heart transplantation: exploring donations after brain death (DBD) and donations after circulatory death (DCD).

The standard procedure for heart transplantation procurement consists of a number of steps involving complex logistic coordination. The procurement process is initiated when a donor is identified at a donor hospital, and the patient has been declared dead. The donor institution then contacts the United Network for Organ Sharing (UNOS) indicating that they have a potential donor. UNOS then screens their list of patients waiting for a heart transplant and contacts the hospital team caring for that patient. The potential recipient institution then initiates a “suitability evaluation”. If the donor's heart is deemed suitable for transplant to the patient selected by UNOS, a team from the recipient hospital is assembled and travels to the donor institution to retrieve the donor's heart. The team then explants the donor's heart and places it in an ice bath for transport to the recipient's hospital. This storage in an ice bath is known as Static Cold Storage (SCS) and has been the standard of care until recently (Figure 1). During SCS the explanted heart does not have a supply of oxygen or nutrients necessary for the generation of energy, which results in ischemic injury. The rationale behind SCS was that hypothermia would minimize the demand for oxygen and essential nutrients, thereby minimizing this ischemic injury. In common medical practice, an effort is made to limit static cold storage time to 3–4 h, beyond which SCS has been shown to increase post-transplant graft dysfunction with attendant medical complications and a higher mortality of the transplant recipient (8–10).

There are several reasons for not utilizing the available hearts, such as age >50 years, female sex, donor comorbidities like cerebrovascular accident, hypertension, diabetes, left ventricle dysfunction, and elevated troponin levels (11). One type of injury that occurs in the hearts during procurement and transportation between donor and recipient for heart transplant is known to be ischemia-reperfusion injury (IRI) (8). IRI leads to complications in the immediate post-transplant phase, which include injury to the right ventricle (RV), left ventricle (LV), lungs, kidneys, and liver. In the immediate post-operative days, recipient patients may require inotropes, pressors and temporary Mechanical Circulatory Support (MCS).

Ischemia-reperfusion injury is a common concern in organ transplantation, especially when the donor heart has been preserved for a period of time, leading to potential damage during the reperfusion phase after transplantation (12).

Depending on the severity of the ischemia-reperfusion injury, a patient might require temporary MCS like an intra-aortic balloon pump or extracorporeal membrane oxygenation (ECMO) to stabilize their blood pressure and cardiac output until the transplanted heart recovers (Short-term mechanical circulatory support (intra-aortic balloon pump, Impella, extracorporeal membrane oxygenation, TandemHeart (13).

While MCS can be helpful in certain situations, it is not routinely needed after every heart transplant and is typically only used when the patient shows signs of significant circulatory instability due to the reperfusion injury (14). These complications and treatments can lead to prolonged CCU stays and more permanent MCS and/or renal replacement therapy. This type of injury can also increase the likelihood of organ compromise and shorten graft survival after discharge. Data also show that this type of injury correlates with a higher incidence of cardiac fibrosis and higher long-term mortality (15–17).

### 2.2 Procurement strategies for reducing the effects of ischemia

The advent of *ex vivo* devices that enable perfusion of hearts during transportation for transplant increases the number of donor hearts available for transplant and provides the possibility of developing new therapies to increase the number of hearts available by preventing ischemic injury, which allows resuscitating and regenerating marginal hearts. The development of OCS®, a device for normothermic perfusion of hearts during transport has been a major step forward towards expanding the number of hearts available for transplant from DCD donors (18). It has also been used to expand the number of donor hearts available from DBD donors by resuscitating marginal hearts (Figure 1) (19). *Ex vivo* preservation models can also be used for the study of new therapies for the failing heart, which could translate to therapies for the treatment of individuals with heart failure. This would be a game changer for the entire field of heart failure.

#### 2.2.1 Static cold storage and devices for transportation prior to implant

**Figure 2:**
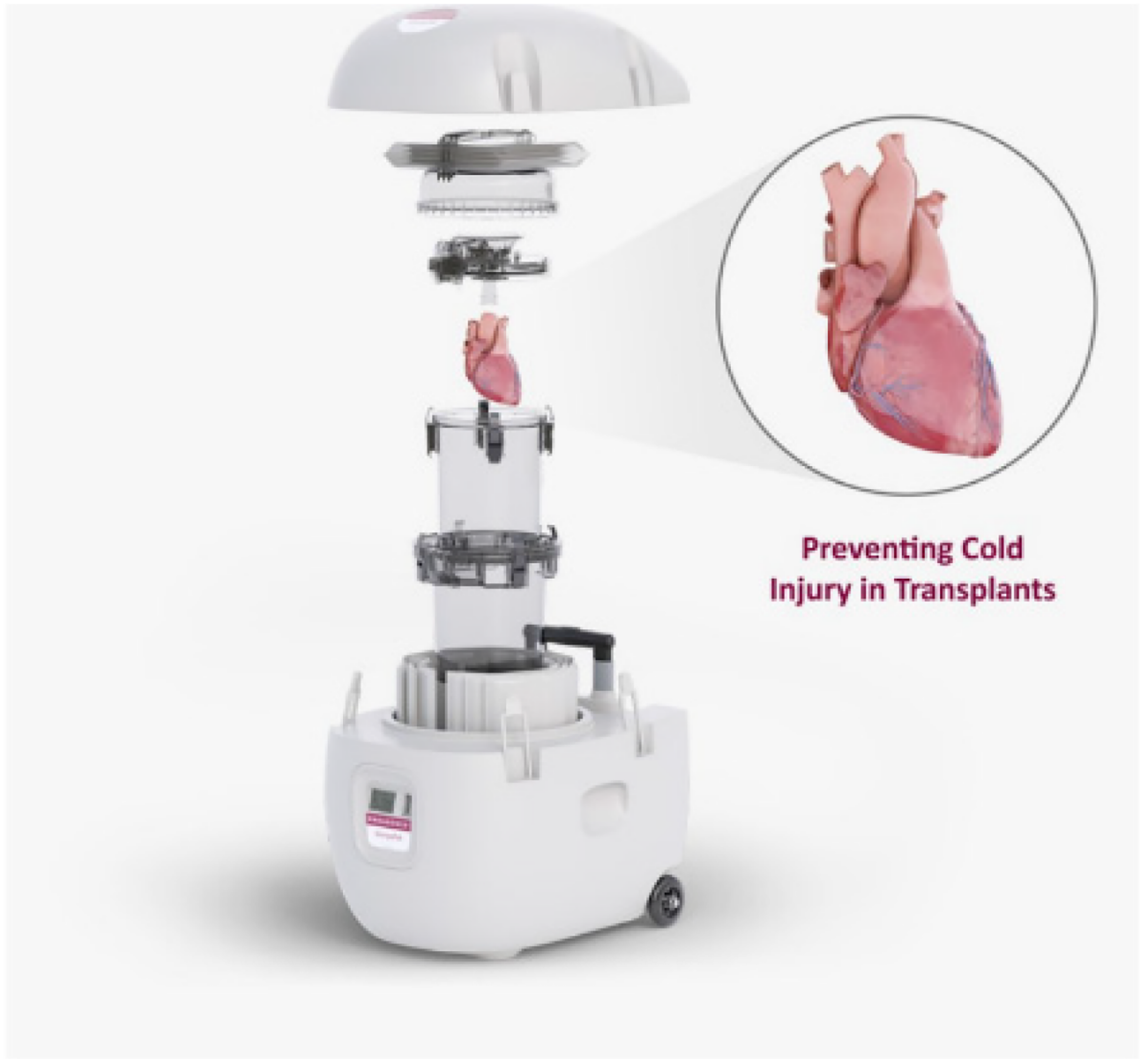
The paragonix sherpapak® cardiac transport system. The heart is submerged in cold cardioplegic solution. The transport system uses proprietary phase change cold packs to maintain temperatures 4–8°C.

Static cold storage has been the traditional heart preservation strategy and standard of care since the first heart transplant and is currently considered a reliable strategy for DBD hearts. It is simple, inexpensive, and able to preserve standard DBD hearts for 3–4 h with acceptable transplantation outcomes (20). But storage at 0**°**C has been found to be less effective than at slightly higher temperatures—called hypothermic storage. Paragonix has developed a controlled hypothermic static storage device called the Shepapak® (Figure 2) that has been approved by the FDA for the transportation of DBD hearts. The Sherpapak® device has been shown to reduce ischemic injury compared to SCS and extend the time for transport of the donor organ. In a pivotal trial, Paragonix has shown that the Sherpapak® can extend the time between explant and implant from the standard of 3 h to 6 h. This has led to a small increase in the number of hearts available for transplant.

#### 2.2.2 Machine perfusion

During static cold storage there is no source of oxygen or nutrients for the heart. Machine perfusion (MP) devices have been developed to address this issue. Perfusion can supply the metabolic needs of the myocardium, thus minimizing irreversible ischemic cell injury and death. For donor hearts, MP can provide a platform to resuscitate, preserve, assess and even possibly recondition the cardiac function prior to planned transplantation. New heart perfusion systems, which are either hypothermic MP (HMP) or normothermic MP (NMP), have been developed that show some success in preserving animal and/or human hearts (21). Systems are already in active clinical use with encouraging outcomes for both DCD and extended-criteria DBD donors (22). They are already altering clinical practice and facilitating the utilization of donor hearts once considered marginal (19).

##### 2.2.2.1 Organ care system (OCS)

**Figure 3:**
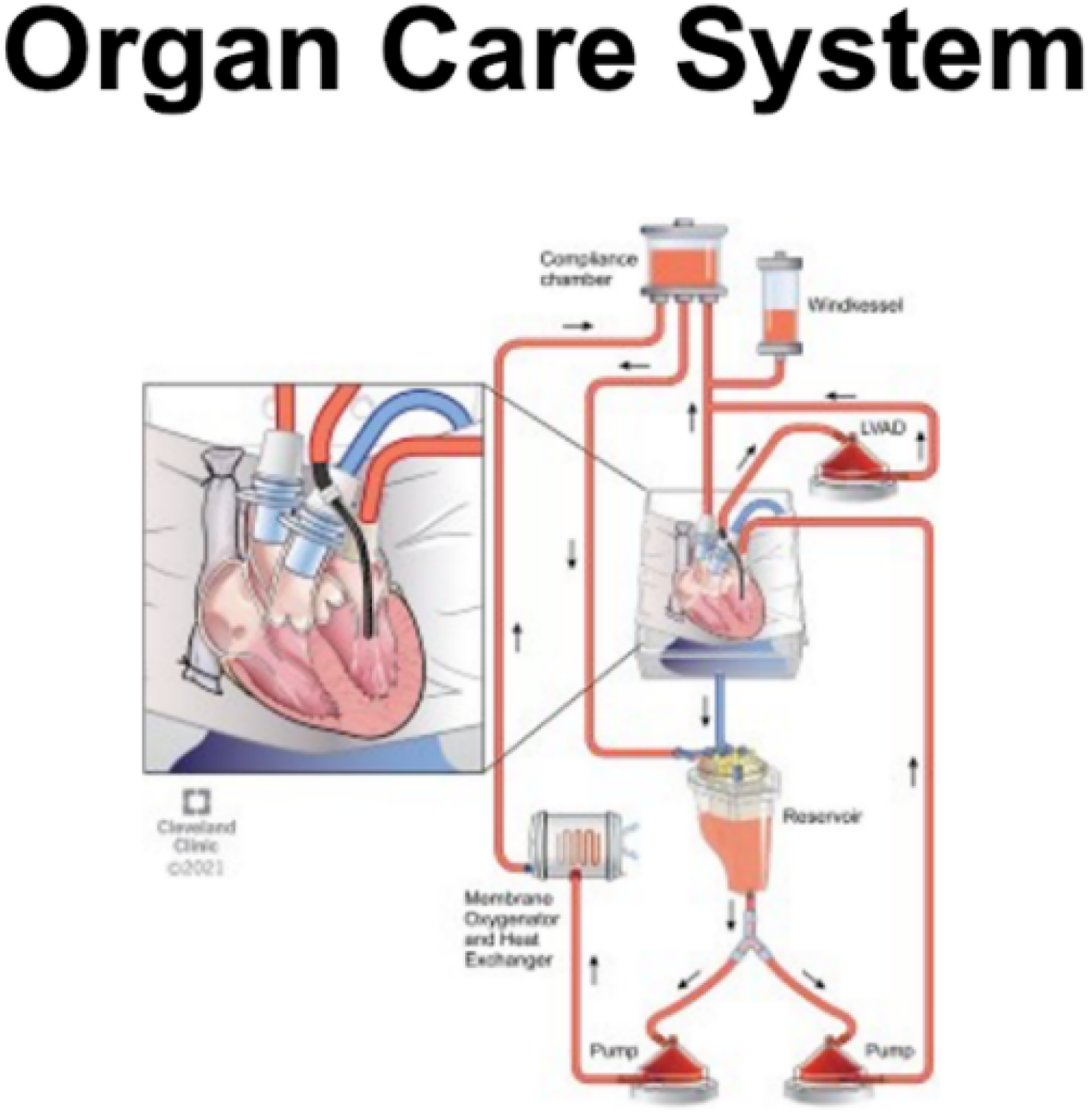
This figure illustrates an organ care system (OCS®) heart machine with the schematic diagram, breaking down the processes involved.

Currently, one commercial perfusion system called the organ care system (OCS®) (Figure 3) is available for clinical use (23, 24). A prolonged safe preservation time allows for the utilization of remote donor hearts and functional assessment allows for the utilization of high-risk donor hearts.

One effective means of expanding the number of hearts available for transplantation is to expand the source by including DCD hearts. Because DCD hearts are more fragile than DBD hearts due to injury inherent in the causes for circulatory death and unavoidable ischemia during the period awaiting declaration of death, the SCS method is not considered the best preservation strategy for DCD hearts, as energy-depleted DCD hearts can hardly tolerate additional cold ischemia (25). In recent years, normothermic *ex vivo* heart perfusion (NMP) has become recognized as a novel strategy for DCD heart preservation. During organ storage NMP can supply donated hearts with oxygenated, warm, and nutrient-enriched blood-based perfusate in a semi-physiological state (26). Therefore, compared with static cold storage, NMP can attenuate myocardial ischemia/reperfusion injury (IRI) injury in DCD hearts, and be used to assess the contractile function of DCD hearts in real-time, prolong storage time, and potentially provide a unique platform for repairing DCD hearts. Moreover, compared with conventional DBD heart transplantation, DCD heart transplantation has provided a similar 30-day and 1-year postoperative survival rate (27, 28). Nevertheless, the inevitable warm ischemia time, which spans from the time systolic blood pressure falls below 50 mmHg after the withdrawal of life-sustaining therapy to the time of reperfusion or cardioplegia, results in more serious myocardial IRI in DCD hearts (29). This injury leads to a higher incidence of primary graft dysfunction (PGD) for the patients undergoing DCD heart transplantation (22, 30, 31).

Transmedics has developed the Organ Care System® (OCS®) (Figure 3), which utilizes normothermic perfusion of the beating heart with oxygenated whole blood. The system is similar to the system of cardiopulmonary bypass used during cardiovascular surgery when the heart is arrested. It has been approved by the FDA for DCD hearts. The use of this technology has increased the number of hearts available for transplant by extending the time for transportation and, therefore, the geographical range for procurement. Since the OCS® device received FDA approval in 2018 for use in transporting DCD hearts, the availability of hearts from DCD donors has steadily increased to 278 transplants in 2022 (32). The OCS® device has shown a one-month mortality rate comparable to that of DBD hearts using static cold storage. The effect on the rate of post-transplant graft dysfunction has not been investigated.

##### 2.2.2.2 Xvivo

More recently Xvivo has developed a device that uses hypothermic perfusion with diluted whole blood in a system like the OCS® device (33). Neither of these devices eliminate injury from ischemia reperfusion, but they can extend the time from explant to implant and make available DCD hearts. Minimizing the ischemia reperfusion injury could increase the time between explant and implant and reduce the rate of post-transplant graft dysfunction and development of fibrosis, which would be of significant benefit. It is important, therefore, to look beyond mechanical strategies to reduce myocardial IRI in donor hearts.

### 2.3 Heart preservation solutions

To mitigate the adverse effects of IRI, a range of heart preservation solutions have been developed, each containing varying concentrations of cellular nutrients, metabolites, electrolytes, and antioxidants. Studies on optimizing perfusate contents and control of resuscitation parameters have produced results that can be used to improve preservation systems for donor hearts (15). The first preservation solution was the Euro Collins solution, formulated in 1960. It was followed by the University of Wisconsin (UW) solution in 1988 (23). Subsequently, due to several modifications to these solutions and the development of new ones, the number of solutions commercially available has grown to more than 150 (34, 35). Currently, the three solutions most used are the HTK solution (Perisoc, Khöler Chemie Pharmaceuticals, Germany), the UW solution (SPS-1, Poland), and the Celsior solution (Institut Georges Lopez, France).

With the advent of normothermic perfusion, it is now feasible to consider an approach to the *ex vivo* recovery or regeneration of diseased organs that combines the use of a machine perfusion system with one of these *ex vivo* therapeutic perfusion solutions. Using this approach with marginal donor hearts, in particular DCD hearts that have been rejected for transplantation, could further expand availability, especially for selected clinical indications (36). Currently, most studies investigating *ex vivo* supported organ reconditioning are executed on an experimental level. In the future, machine perfusion combined with *ex vivo* therapeutic perfusion may eventually allow the treatment of diseased hearts either *in situ* or *ex vivo* followed by autologous reimplantation thereby reducing the number of patients in need of organ transplantation (37–39).

Therapies such as standard cardioplegic solutions (40), antioxidants (41), and anti-inflammatory approaches (42) have been researched to optimize recovery and utilization of donor hearts. We present in section 3.8.1 below a novel therapeutic approach that uses peptide mimetics of BMP-7 to activate the BMP signaling pathway in order to inhibit inflammation, apoptosis, and fibrosis.

### 2.4 Metabolic and other markers of ischemia

*Ex vivo* machine perfusion provides the opportunity to not only evaluate a donor heart for implantation but also offers the possibility of rehabilitating an injured donor heart. Following metabolic and biochemical markers in the perfusate is the primary, and non-invasive, mode of evaluation. For perspective on how the health of a heart is evaluated during *ex vivo* machine perfusion, we review here what happens at the cellular level during ischemia.

The lack of oxygen shuts down oxidative metabolism, the Krebs cycle, which takes place in the mitochondria. As long as glucose is present, glycolysis continues producing two ATP and two pyruvate molecules per molecule of glucose. But compared to the approximately 32 ATP effectively produced per molecule of glucose by the complete oxidative metabolic pathway, the absence of oxygen results in a major decrease in metabolic energy production. In the ischemic state with oxidative metabolism shut down, pyruvate produced by glycolysis cannot enter the blocked Krebs cycle. Without a means to remove the pyruvate, glycolysis would also be shut down and the two ATPs that it generates lost. Instead, pyruvate is converted to lactate and excreted from the cell. During SCS, the excreted lactate builds up in the interstitial spaces of the organ tissue and diffuses into the perfusate. Because lactate is in equilibrium with lactic acid, depending on the buffering capacity of the perfusate, increasing lactate concentration results in lowered pH and the danger of acidosis damage to the tissue (43). The CORI cycle, which is located primarily in the liver, is the metabolic pathway that converts lactate back to glucose, at the expense of 6 ATP. During oxidative machine perfusion of the heart, the lactate concentration in the perfusate will not decrease, due to the lack of tissue containing the CORI cycle, but neither should it increase due to resumed oxidative metabolism. Any increase is indicative of a source of continued ischemia and of tissue damage due to damaged mitochondria (44). Metabolic markers of improved heart health include the rate of O_2_ and glucose use—difference in O_2_ and glucose concentrations before and after the heart container—and the rate change of lactate concentration.

Under ischemic conditions or when glycogen and glucose concentrations are low, the heart switches to a different source of energy, metabolism of free fatty acids (45). Fatty acids must first be activated by conversion to thioesters, an O_2_ dependent process. Activated fatty acids are then converted to acetyl-CoA by beta-oxidation, a process that is not dependent on O_2_. Acetyl-CoA can directly enter the citric acid cycle to produce significant amounts of ATP. Under ischemic conditions where the citric acid cycle has shut down due to lack of O_2_, fatty acid metabolism ends with acetyl-CoA. In their review, Jaswal et al. (45) note that upon reperfusion “fatty acid β-oxidation also rapidly recovers, leading to an inhibition of pyruvate dehydrogenase and an increased production of lactate and protons” before oxidative metabolism in mitochondria has recovered.

Conditions or treatments affecting circulating free fatty acids may impair myocardial metabolism during hypoxia, ischemia, and reperfusion (46). Fatty acid oxidation is significantly reduced in the ischemic heart compared to a healthy heart due to the ischemic damage sustained during the warm ischemic period prior to procurement, leading to impaired mitochondrial function and reduced ability to utilize fatty acids as a primary energy source.

Upon initiation of *ex vivo* perfusion, a DCD heart will exhibit low rates of fatty acid oxidation due to the metabolic disruption caused by warm ischemia (47). As the perfusion progresses, a significant metabolic shift occurs where the heart switches from primarily utilizing fatty acids as an energy source to relying more heavily on glucose and lactate, a phenomenon often referred to as a “metabolic switch” due to the altered conditions of ischemia and reperfusion experienced by the DCD heart (48).

Other cellular reactions to ischemia are oxidative stress, inflammation, and apoptosis, which are evidenced by specific biochemical markers such as: HNE (oxidative stress); increased expression of TNF-α, IL-6, NF-κB, and MPO (inflammation); DNA strand breaks, increased expression of Bax, cleaved caspase-3, cytochrome C, and decreased expression of Bcl2 (apoptosis); and expression and secretion of cytokines, chemokines and adhesion molecules (pro-inflammatory markers). Upon organ implantation into a recipient, proinflammatory cytokines, such as IL-1 and TNF-α, and chemokines are produced within hours of reperfusion in grafts. Chemokines mediate early migration of neutrophils and macrophages into the graft (49, 50). Inflammatory activation of graft endothelium (51), platelets, the coagulation cascade, and the complement (52) play important roles in early graft injury and subsequent graft vasculopathy. This response is separate and distinct from immune rejection of the graft.

### 2.5 *Ex vivo* therapeutic interventions

*Ex vivo* perfusion systems present a significant opportunity to serve as a development platform for the administration of therapeutic interventions on donor hearts without causing adverse effects on other donor or recipient organs. The central objective of these interventions is to mitigate the impact of IRI, with a particular focus on the potential to rejuvenate marginal organs for viable transplantation. Although these therapeutic applications are still in the preclinical phase and have not yet undergone clinical testing, their preliminary outcomes are promising. The prospective integration of *ex vivo* therapies has the potential to not only enhance graft viability, but also contributes to expanding the existing donor organ pool, a prospect that has significant potential for the field of transplantation.

Several potential therapies, such as standard cardioplegic solutions (40) antioxidants (41) and anti-inflammatory approach (42) have been studied to optimize recovery and utilization of donor hearts.

Following a cardiac ischemic event, key cellular effects that are targeted by current cell transplantation or therapeutic strategies include: mitochondrial dysfunction (53), oxidative stress (54), cell death pathways (apoptosis, necrosis, autophagy) (55), inflammation (56), and extracellular matrix remodeling/fibrosis (57), with a focus on restoring damaged cardiomyocytes and promoting angiogenesis to revascularize the affected area. Cardiomyocyte-like cells derived from embryonic stem cells or induced pluripotent stem cells (iPSCs): aim to replace damaged cardiomyocytes with functional ones (58). Mesenchymal stem cells (MSCs) possess paracrine effects, promoting angiogenesis, anti-inflammatory activity, and tissue repair (59). Endothelial progenitor cells can contribute to new blood vessel formation, improving blood flow to ischemic regions (60).

#### 2.5.1 Therapeutic interventions employing stem cells and microRNAs

Cardiac regeneration and functional recovery after myocardial ischemic injury has been the focus of much study but achievement of this goal remains elusive (4, 61). In mammals, heart injuries such as those induced by myocardial ischemia and myocardial infarction (MI) result in substantial loss of cardiac muscle cells (cardiomyocytes), which are replaced by fibrotic scar tissues. This condition coupled with the very limited regenerative capacity of the adult mammalian heart often leads to heart failure (HF) (62, 63). Finding targets for the development of drugs or harnessing the potential of stem cells has fostered significant scientific endeavor and produced marked strides towards understanding the mediators of the processes involved in myocardial injury (64–69). A great deal of this effort has focused on using stem cells to regenerate cardiac tissue.

A promising therapeutic strategy for cardiac regeneration following myocardial ischemia and infarction is to initiate myocyte regeneration through the transplantation of stem cells. However, the major challenge for the development of such therapies is the limited survival and function of transplanted stem cells (70, 71). Two main cell types are currently under investigation in heart repair from ischemic injury. Mesenchymal stromal cells (MSCs) indirectly support endogenous regenerative capacities after transplantation. Induced pluripotent stem cell-derived cardiomyocytes (iPSC-CMs) directly contribute to the restoration of function by integrating into the damaged myocardium. Intramyocardial or intracoronary delivery of MSCs significantly reduced cardiac scar size after myocardial ischemia in a porcine model (72–74). Recent studies have shown that intravenously injected MSCs can improve myocardial IRI in a porcine model by preventing microvascular obstruction (75). Aggregates of iPSC-CMs, which are termed cardiac spheroids, have been developed to improve engraftment (76). The transplantation of iPSC-derived cardiac spheroids is safe and effective for improving cardiac function in rat and swine HF models (77).

Despite the promising outcomes of stem cell treatment against ischemic heart disease in previous experimental and clinical studies, current evidence supports neither the incorporation of the infused stem cells into the injured myocardium, nor their *in vivo* differentiation into functional myocytes (78, 79). The observed stem-cell-mediated benefits have been shown to be attributable to the paracrine functions of stem cells. When cardiomyocytes (CM) derived from human induced pluripotent stem cells (hiPSC) are injected in the cardiac tissue, human iPSC-CMs have longer action potential and lower cell-to-cell coupling than adult-like CMs (80). The electrophysiological properties of these immature iPSC-CMs generate electrophysiological gradients that favor arrhythmias (81, 82).

Recent evidence suggests that small extracellular vesicles, called exosomes, appear to play a key role, particularly because they contain microRNAs that have been found to have clinical potential in the treatment of ischemic heart disease (83–85). Although most studies refer to microRNAs as being responsible for the therapeutic role of extra cellular vesicles (EVs) or exosomes, proteins have been reported to contribute as well. For instance, cardiac progenitor cells (CPC)-EVs administrated in the heart 1 h after ligation reduced CD68 + macrophages in the treated rats one month after EV injection (81). Pregnancy-associated plasma protein-A (PAPP-A) was found to be responsible for the therapeutic effect. PAPP-A is a protease responsible for the cleavage of insulin growth factor-1 (IGF-1) binding protein-4, which transports IGF-1. Once released from its complex, IGF-1 can act as an immunomodulator in the heart (82).

Considering the limited availability of autologous stem cells, exosomes derived from their allogeneic and xenogeneic counterparts may provide therapeutic advantages (86). Nevertheless, there are several challenges and shortcomings, such as observed substantial heterogeneity among studies, no significant differences emerged in mortality and ventricular arrhythmia risk in iPSC-CM treatment vs. controls, that need to be abridged before stem cell therapy becomes a norm in clinical settings. A large clinical trial of transendocardial mesenchymal precursor cells in patients with heart failure did not reach statistical significance for its primary and secondary endpoints (87).

Recent animal studies have demonstrated that Normothermic EVHP (NMP) combined with conditioned medium treatment derived from bone marrow mesenchymal stem cells (BMSCs) can alleviate warm IRI in the DCD animal models (88). A 25-min warm ischemia injury significantly increased the level of oxidative stress (increased expression of HNE), inflammation (increased expression of TNF-α, IL-6, NF-κB, and MPO), and apoptosis (increased expression of Bax, cleaved caspase-3, Cytochrome C and DNA strand breaks, and decreased expression of Bcl2) in the DCD hearts. Compared with the DCD-control group, normothermic *ex vivo* heart perfusion combined with CM treatment decreased the level of oxidative stress (decreased expression of HNE), inflammation (decreased expression of TNF-α, IL-6, and NF-κB), and apoptosis (decreased expression of Bax, Cleaved caspase-3, Cytochrome c, and DNA strand breaks) in the DCD heart after *ex vivo* heart perfusion, which might alleviate the shortage of donor hearts by adopting DCD hearts (88).

*Ex vivo* perfusion systems can facilitate the delivery of therapeutic cells, such as stem cells or genetically modified cells, directly to the donor heart. These cells have the potential to promote tissue repair, regeneration, and immunomodulation, further enhancing the heart's viability, or for example, human multipotent stromal cells (89, 90). Recent studies have demonstrated that Normothermic EVHP combined with conditioned medium treatment derived from bone marrow mesenchymal stem cells (BMSCs)) can alleviate warm IRI in the animal models of DCD hearts. Although these strategies have shown great promise in preclinical studies, it is important to note that translating them into clinical practice requires rigorous testing and validation.

#### 2.5.2 Therapeutic interventions employing genetically modified cells

Gene transfer techniques can be utilized to introduce specific genes into the donor heart during *ex vivo* perfusion in animal models. These genes can encode protective factors or enzymes that counteract IRI processes. By isolating the heart in a metabolically and immunologically favorable environment and preventing off-target effects and dilution, it is possible to directly control factors that enhance the success rate of cardiac gene therapy. Gene therapy research during normothermic *ex vivo* heart perfusion has involved adenovirus mediated gene transfer (91–96) using rabbit or porcine hearts. One study investigated adeno-associated viral (AAV) mediated gene transfer in porcine hearts (97). The most important finding was the ability to achieve durable transgene expression using AAV-mediated gene transfer for up to 35 days following heterotopic transplantation, without signs of systemic off-target expression, rejection, or inflammation in the graft. To date, local immunomodulation and enhanced myocardial tolerance to ischemia-reperfusion injury have been achieved using gene transfer during *ex vivo* heart perfusion in rodent models (98). Future studies will need to focus on replicating these findings in large animal models and humans.

#### 2.5.3 Therapeutic interventions employing metabolic modulation

Manipulating the metabolic pathways within the donor heart during *ex vivo* perfusion could promote energy production and reduce the negative consequences of metabolic disruption during IRI. By restoring oxygenation and providing metabolic substrates, machine perfusion potentially allows for the correction of metabolic derangements caused by IRI (99).

#### 2.5.4 Therapeutic interventions employing pharmacological agents

Targeting specific candidates implicated in organ IRI is challenging due to the complex molecular pathways that are activated. Some of the activated pathways and molecules include the complement cascade, the innate immune response and toll-like receptors (TLRs), CD4T lymphocytes, inflammatory cytokines propagating the post-inflammatory response, nuclear factor κB (NF-κB) leading to production of TNF-α, adhesion molecules, apoptotic pathway activation, and reactive oxygen species (ROS) production and release (100, 101). Pig liver studies rely on using a combination of therapies that block multiple, perhaps redundant, ischemic/reperfusion injury pathways in order to achieve a significant reduction in injury and overall improvement in graft function (102). Various pharmacological agents, such as antioxidants, anti-inflammatory drugs, and vasodilators, have been administered to the donor heart during *ex vivo* perfusion in animal models. These agents aim to counteract oxidative stress, reduce inflammation, and improve vascular function, ultimately protecting the heart from IRI-related damage.

#### 2.5.5 Therapeutic interventions employing growth factors

Various growth factors such as fibroblast growth factor 1 (FGF1) (103), vascular endothelial growth factor (VEGF) (104), bone morphogenetic protein-2 (BMP-2) (105), BMP-10 (106), and systemic factors like thyroid hormones (107) and glucocorticoids (108) have been shown to modulate dedifferentiation and proliferation of endogenous cardiomyocytes, thereby potentially modulating heart regeneration in adult mammals. In a recent study, Vukicevic (109) found that bone morphogenetic protein 1.3 (BMP1.3) levels were elevated in both patients and animal models of myocardial infarction. In a cardiac fibrosis animal model, treatment with a specific monoclonal antibody against BMP1.3 alleviated cardiac fibrosis, reduced collagen deposition and cross-linking, and was paralleled by enhanced cardiomyocyte survival both *in vivo* and in primary cultures of cardiac cells. Mechanistically, the monoclonal antibody against-BMP1.3 has the effect of inhibiting the TGF-beta pathway, thereby reducing myofibroblast activation and inducing cardio protection through BMP-5. BMP-7 administration stimulated mouse cardiomyocyte cycling at postnatal-day 7, when cardiomyocytes normally cease to proliferate, and enhanced cardiomyocyte regeneration with functional recovery *in vivo* in adult mice following myocardial infarction (110).

### 2.6 Therapeutic intervention by blocking inflammatory, apoptotic and necrotic pathways

Anti-inflammatory agents can enhance the protective effect of *ex vivo* dynamic organ preservation by further curbing the inflammatory response during perfusion. In a recent study, a warm ischemic time of 25 min significantly up-regulated the levels of IL-6, TNF-α and NF-κB, an inflammatory response in the DCD heart submitted to EVHP, while treatment with melatonin (N-acetyl-5-methoxytryptamine) led to a significant decrease in the expression of IL-6, TNF-α, and NF-κB in the DCD heart (111).

**Figure 4:**
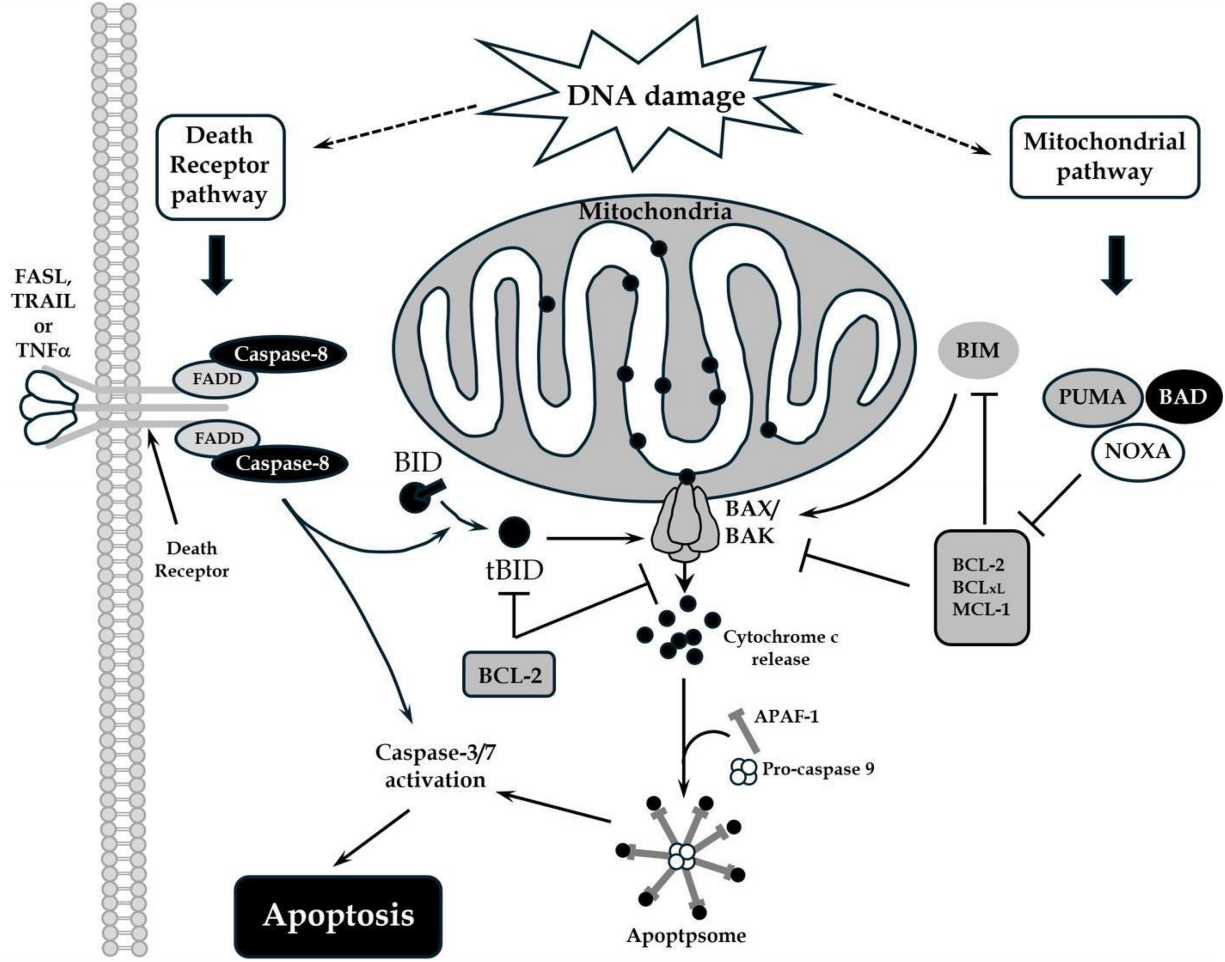
The death receptor and mitochondrial pathways of apoptosis: DNA damage triggers the death receptor and mitochondrial apoptotic pathways. Death ligands that bind to their respective receptors trigger the recruitment of adaptor molecules (FADD), and subsequent recruitment and activation of caspases to mediate apoptosis. Activated caspases can also cleave BID to promote the mitochondrial pathway of apoptosis. This pathway is defined by mitochondrial outer membrane permeabilization (MOMP) and is regulated by the BCL-2 proteins. Pro-apoptotic proteins such as BID or BIM promote BAX or BAK homo-oligomerization in the mitochondrial membrane, whereas anti-apoptotic proteins such as BCL-2 and BCL-xL inhibit this process. De-repressor proteins BAD, PUMA or NOXA bind the anti-apoptotic proteins and reduce the threshold for BAX/BAK activation. MOMP results in cytochrome c release into the cytosol, which promotes APAF-1 oligomerization, caspase activation, and apoptosis.

Myocardial apoptosis involves both the death domain receptor and mitochondrial pathways (Figure 4). Both pathways converge on caspase activation (112). The death receptor pathway is extrinsic, involving cell surface receptors. The mitochondrial pathway is intrinsic, using mitochondria and the endoplasmic reticulum. Evidence indicates that the death receptor and mitochondrial pathways are not isolated systems. Instead, significant crosstalk and “biofeedback” regulates the apoptotic machinery (113). Interception of the apoptosis pathway in porcine hearts using hypothermic perfusion solutions infused with small interfering RNA molecules targeting key apoptotic and inflammatory enzymes has had notable outcomes in diminishing cellular apoptosis and mitigating myocyte injury. Furthermore, this approach has resulted in enhancement of donor myocardial function (114, 115). In a distinct porcine transplantation model, the introduction of oxygen-derived free radical scavengers into the perfusion process has shown the potential to enhance graft functionality while concurrently ameliorating cellular edema (116).

Yet another preclinical investigation unveiled the efficacy of a therapeutic regimen that couples two inhibitors of the necrosis pathway, resulting in a discernible reduction in IRI among rat cardiomyocytes (114, 117). Therapies that foster angiogenesis have also garnered attention. Examples encompass interventions involving the inclusion of vascular endothelial growth factor (VEGF) (118) and prokinectin receptor-1 (89). These interventions have manifested the capacity to elevate the survival rate of cardiomyocytes subjected to ischemia.

Melatonin, which is mainly synthesized by the pineal gland in mammals, is regarded as an antioxidant (89), anti-inflammatory (119), and anti-apoptotic (120) molecule. It has been shown that the combination of normothermic EVHP and melatonin post-conditioning could be a novel and promising donor heart preservation strategy, which could ameliorate myocardial IRI and improve cardiac function of DCD hearts, thereby increasing the number of transplantable hearts in heart transplantation (111). Given that inflammation and apoptosis lead to the development of fibrosis, Hosseinzadeh et al. (121) have proposed that melatonin could also protect against fibrosis. Below we describe a growth factor mimetic that has been shown to protect against the development of fibrosis and to reverse fibrosis (122).

### 2.7 Bone morphogenetic proteins (BMPs)/signaling

Several other therapies have been investigated for the treatment of ischemia-reperfusion and advanced decompensated heart failure (123). Most of these therapies targeted pathways downstream in the pathologic processes. As such, they affect only a single arm of a multifaceted process and are therefore limited in their beneficial effects. By looking upstream to targets that have broader effects, we have discovered agents that are safe and block the multifaceted cardiac cellular injury (124). Our target is a bone morphogenic growth factor (BMP) that belongs to the TGF beta superfamily.

BMP-7 signaling exerts important actions on fibroblasts, cardiomyocytes and macrophages. Anti-fibrotic effects of BMP-7 have been reported in many systems and may be mediated, at least in part, through suppression of collagen synthesis by cardiac fibroblasts (125) and through inhibition of endothelial to mesenchymal transition (EndMT) (126). In cardiomyocytes, BMP-7/signaling attenuates hypertrophy by inhibiting TGF-*β* responses (125). In macrophages, BMP-7 has been reported to promote M2 polarization (127). Considering the absence of endogenous BMP-7 induction in infarcted and remodeling hearts, administration of exogenous BMP-7 has been suggested as a potential strategy to attenuate fibrosis and adverse remodeling.

We have targeted a growth factor that belongs to the TGF beta superfamily, a developmental pathway critical to the formation of organs during embryogenesis. Bone morphogenic protein 7 (BMP-7) is involved in embryogenesis, development and the maintenance of adult tissue homeostasis, and is a potent antagonist of TGF beta action (128–130). It can block TGF beta-induced inflammation and apoptosis, and block/reverse TGF beta-induced fibrosis (126, 131).

The transforming growth factor β (TGF-β) superfamily consists of a large group of pleiotropic cytokines, including TGF-βs, activins, and bone morphogenetic proteins (BMPs), which are critically involved in embryogenesis, development and the maintenance of adult tissue homeostasis. Alterations in BMP signaling pathways often result in severe human diseases (132, 133) including cardiovascular diseases (134). BMP canonical signaling in target cells involves type I and type II receptors and the formation of tetrameric receptor complexes. BMPs preferentially bind type I receptors and recruit type II receptors. The constitutively active type II receptor trans-phosphorylates and activates the type I receptor upon forming the heterotetrameric receptor complex. Activated BMP type I receptors (BMPR-I), often referred to as activin-like kinases (ALKs), have serine/threonine kinase activity. Their canonical cell effectors are the phosphorylated SMAD1/5/8/9 transcription factors (132, 135).

There are also three type II receptors involved in BMP signaling: BMPR-II and activin type II receptors ActR-IIA and ActR-IIB (136–138) while TGF β interacts with the type I receptor (ALK5) and type II receptor (TGFR-II). Activin binds type I receptors ActR-IA (ALK2) and ActR-1B (ALK4) and type II receptors ActR-IIA and ActR-IIB.

**Figure 5:**
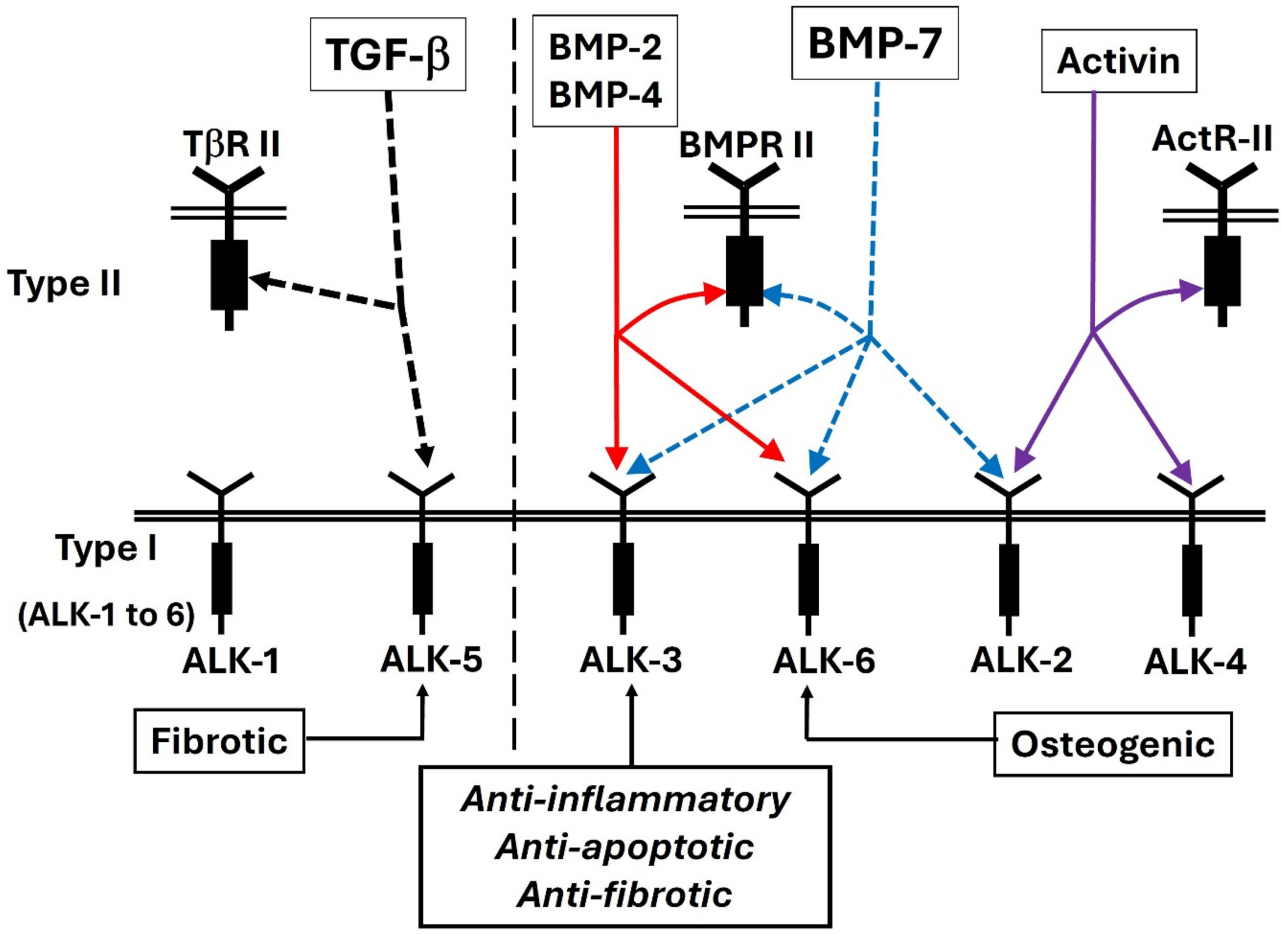
The TGF beta/BMP/activin pathways are mediated by their specific type I and type II receptors. BMP binds any of three type I receptors: BMPR-IA (ALK3), BMPR-IB (ALK6) and a Type IA activin receptor ActR-IA (ALK2) (138–140).

**Figure 6:**
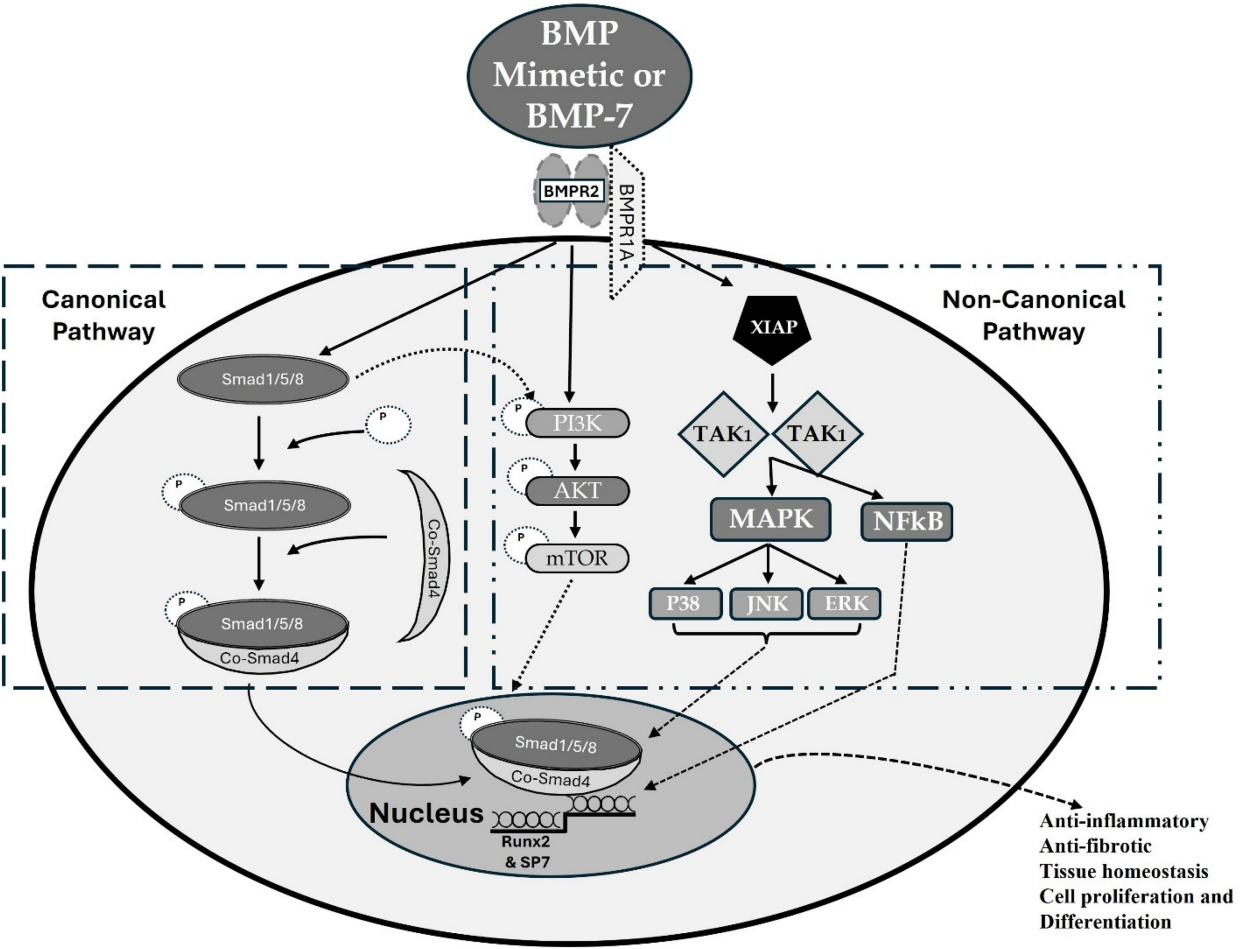
BMP signaling pathways. BMP mimetic/BMP-7 transduces signals in target cells by binding to a specific membrane bound receptor BMPR-II and phosphorylates BMPR-I, which activates both the canonical and the non-canonical pathways. In the canonical pathway, activated BMPR-II leads to phosphorylation of Smad-1/5/8 which complexes with Smad-4 and translocate the signal. In the non-canonical pathway, p38, Mitogen-activated protein kinase (MAPK), c-Jun-N terminal Kinase (JNK), ERK and NFKB were activated via the activation of X-linked inhibitor of apoptosis protein (XIAP), TGF-beta activated kinase 1 (TAK1) and TAK1 binding protein (TAB1) whereas PI3K/Akt were activated by both BMPR-II and Smad-1/5/8. Altogether, this influences the different transcription factors and regulates the gene expression.

BMP-7 binds BMPR-IA (ALK3), BMPR-IB (ALK6), and the activin A receptor type 1 ACVR-I (ALK2) (Figure 5). BMP activates both canonical and non-canonical pathways (Figure 6). In the canonical pathway, it activates BMPR-II, which leads to phosphorylation of Smad-1/5/8, which then complexes with Smad-4 and transmits the signal. In the signaling pathway involving XIAP, TAK1, and TAB1, the receptor complex is typically the first to be activated upon ligand binding, and then it triggers the downstream activation of the TAB1-TAK1 complex, with CIAP playing a role in modulating this activation by interacting with TAB1, not being directly activated by the receptor itself (141, 142).

Altogether, this activation influences the different transcription factors and regulates the gene expression. Notably, several reports demonstrate the involvement of BMPR-IA mediated signaling in the protective role of BMP-7 against renal fibrosis (126, 143).

Recent preclinical investigations have demonstrated that endogenous and exogenous rhBMP-7 protect the myocardium against maladaptive phenotypic plasticity in experimental models of clinically relevant heart diseases, which strongly suggests the cardioprotective therapeutic potential of BMP-7-based approaches (144–148).

The cell-type differential bioactivity of BMPs is dependent on the receptors expressed (149). Therefore, whether BMPR-IA signaling may be involved in the cardioprotective effects of BMP-7 was explored. It was observed that BMPR-IA (BMP type 1 receptor a, ALK3) was the most abundantly expressed receptor in the healthy LV myocardium in humans and mice; on the other hand, ACVR1 (Activin type I receptor) was scarcely expressed, and BMPR-IB (BMP type 1 receptor b, ALK6) was virtually absent. The expression of Smad proteins in infarcted or fibrotic myocardium is different from that in the normal myocardium. No matter whether it is assessed in early or late-stage cardiomyopathy, the selective expression of Smad proteins is correlated with cardiac fibrosis and elevated collagen synthesis levels (150, 151). The expression of Smad 2, 3 and 4 is upregulated at the infarct scar as well as in the peri-ischemic border zone (151) and is closely correlated with increased collagen type I expression (152). Crosstalk exists between BMP and TGF-β in their regulation of different signaling pathways. BMP receptor-ligand (e.g., BMP-7) binding can activate Smad1/5/8 signaling and induce the expression of inhibitors of differentiation 2 and 3 (ID2 and ID3). ID2 and ID3 can prevent Smad2/3 phosphorylation and thus counteract TGF-β/Smad signaling (153).

The BMPs have some potentially beneficial therapeutic effects on cardiac development and anti-apoptosis by acting on the Smad proteins. BMP-2 may have therapeutic potential for curing chronic myocardial ischemia by improving the contractility of cardiomyocytes and preventing cardiomyocyte cell death (105). Injection of BMP-2 reduces the infarct size in mice in a left anterior descending artery ligation model. Mice treated with BMP-2 are characterized by reduced cardiomyocyte apoptosis rates. BMP-7 gene therapy limited pathological remodeling in the diabetic heart, conferring an improvement in cardiac function (154). The clinical use of rhBMP-7 in tissue-engineered products has been approved by US and EU agencies to induce localized osteogenesis in orthopedic and maxillofacial applications (155).

### 2.8 BMP mimetics

The important step in developing a BMP-7 mimetic is first and foremost that of separating out the osteogenic activity that has been shown to lead to the formation of ectopic bone when treating soft tissue injury. Furthermore, the whole BMP-7 protein is subject to inhibition by natural inhibitory proteins such as follistatin, chordin and gremlin (156). A mimetic would likely be immune to this inhibition. Small peptide mimetics are also less likely than biologics to induce an immune response. Peptide mimetics of 20 residues or less are synthesized chemically, which is a much simpler, cheaper, and reliable form of manufacturing than fermentation, the primary method for producing biologics. Any potential therapeutic benefit of restoring BMP-7 function using systemic rhBMP-7 is hampered by bioavailability, induction of neutralizing autoantibodies against BMPs, and a range of potential adverse effects (157).

There have been several publications describing efforts to design and test BMP mimetics (see Table 1). Most of these efforts have found compounds that are osteoinductive and, therefore, have limited applications to the treatment of diseases affecting soft tissues such as the heart, the lung, the liver, the kidney, the pancreas, or cancers (159–161). One compound has been found that is effective in cell-based assays indicating it could be used to treat pulmonary hypertension (162). The compound described is not a true mimetic since it cannot act alone, but rather enhances the effects of BMP-9. Other efforts to find BMP pathway agonists using high throughput screening have had limited success (163–166).

**Table 1:** BMP agonists/mimetics.

BMP agonist/BMP mimetic	Derived/designed from type of BMP/region	Target outcome	Reference
A cyclized BMP-7 derived peptide, THR-123	Covers the beta turn that is C-terminal to the “knuckle” of BMP-7 (see Figure 7C)	Reversed established kidney fibrosis in mouse models of chronic renal injury	(149)
A cyclized BMP-7 derived peptide, THR-123	Covers the beta turn that is C-terminal to the “knuckle” of BMP-7 (see Figure 7C)	Induced nongenetic conversion of human pancreatic exocrine cells to insulin-expressing and Functional (glucose-responsive) endocrine cells with a capacity for rapid reversal of diabetes *in vivo*	(158)
A cyclized BMP-7 derived peptide, THR-123, THR-184	Covers the beta turn that is C-terminal to the “knuckle” of BMP-7 (see Figure 7C)	Function as agonists of BMPR-IA (BMP type I receptor), attenuated overexpression of remodeling-related genes and alleviated LV dysfunction in aortic stenosis	(122)

**Figure 7:**
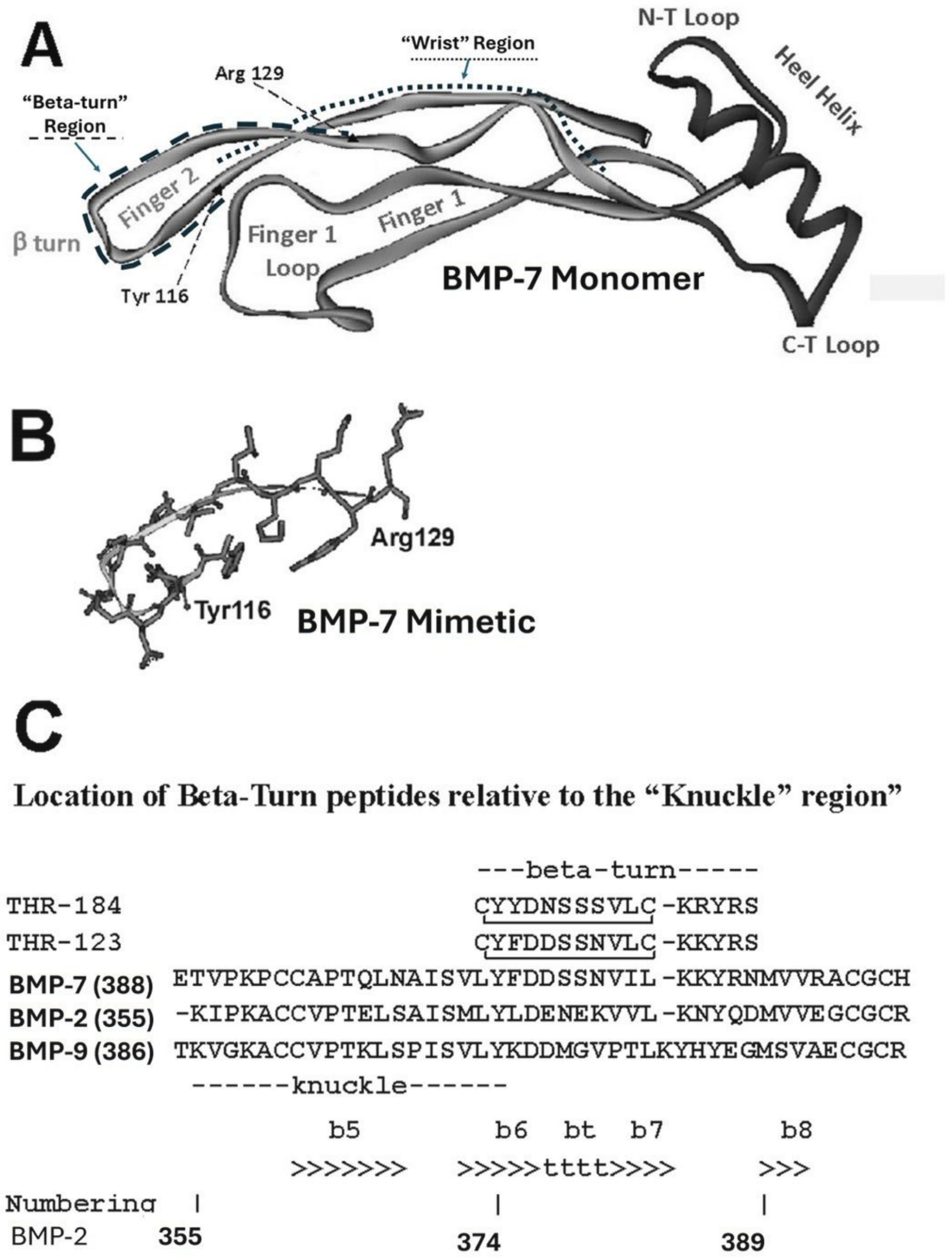
Structure diagrams of the BMP-7 monomer and the region covered by the mimetic. **(A)** Ribbon diagram showing the secondary structure of the BMP monomer, which contains three structural regions: antiparallel beta sheets of “Finger 1” (with the large terminal loop), “Finger 2” (with the tight beta-turn), and the “Heel” alpha helix. Initial targets for mimetic development were the terminal loops of fingers 1 and 2, loops at the C-terminal, and N-terminal loops at the ends of the heel helix. **(B)** The region around the beta turn of finger 2 proved to have activity similar to BMP-7 and became the lead for further mimetic development. **(C)** The “beta-turn” region covered by the mimetic is immediately C-terminal to the “knuckle” region covered by other BMP mimetics. Residue position numbers are based on BMP-2 residue numbers. Secondary structure: beta sheet (>>>>), segments of which are labelled e.g., “b6”; beta turn (bt, tttt). Peptide disulfide bond: C C.

In recent years, studies have advanced our knowledge of the cellular and systemic functions of the BMPs. Therapeutics derived from BMPs have been developed for the treatment of diseases such as cardiovascular and kidney diseases (149, 167–169). The identification of BMP mimetics or BMP agonists remains an attractive strategy (see Table 1). Peptides that are designed from the knuckle region (Figure 7C) of BMP-2, BMP-7 and BMP-9 have been found to display osteogenic activity like the corresponding BMP molecule (159–161, 170). Peptides designed based on the BMP-9 knuckle region have been shown to induce differentiation of murine preosteoblasts (MC3T3-E1 cells) and induce cholinergic differentiation in human SH-SY5Y neuroblastoma cells (171, 172). Furthermore, peptides from the prodomain of BMP-7, namely BFP-1/2/3, have also been shown to induce stronger alkaline phosphatase activity in multipotent bone marrow stromal cells (MBSCs) (173–175). In addition, a BMP-2 knuckle peptide has been shown to possess similar bone inducing activity *in vivo* but did not induce the side effects observed with BMP-2 (176).

BMP mimetic peptides have the potential for improving specificity. For example, peptide P3 designed from the “wrist area” of BMP-9 enhanced BMP-9-induced Smad1/5 phosphorylation selectively in human pulmonary artery endothelial cells (hPAECs) but inhibited BMP-4-induced Smad1/5 phosphorylation in human dermal microvascular endothelial cells (HMEC-1) (164).

Although these findings are exciting, very few studies have performed in-depth binding and mechanistic investigation of how these peptides achieve their specificity.

#### 2.8.1 THR-123 & THR-184

**Figure 8:**
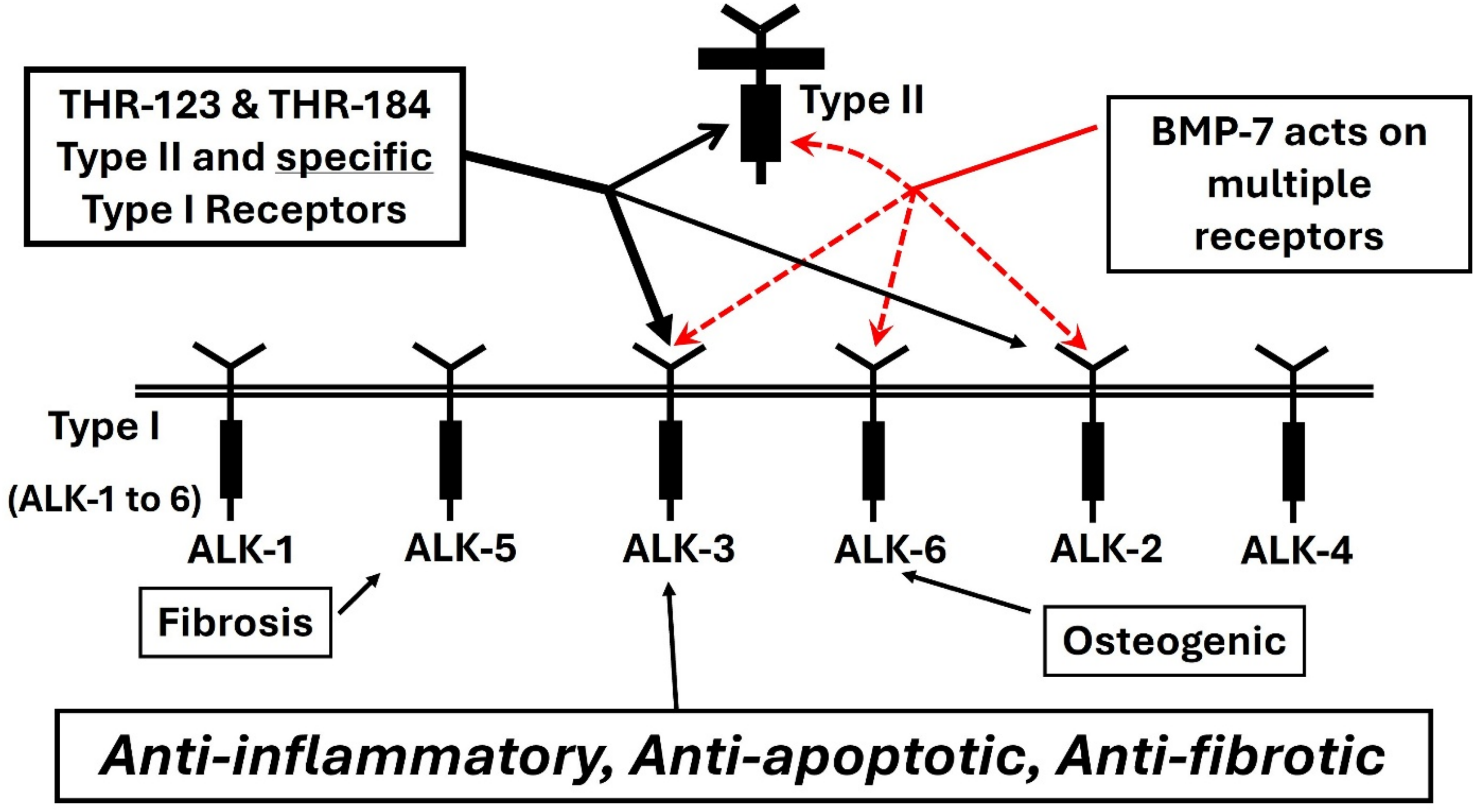
THR-123 and THR-184 binding like BMP-7, BMP mimetics THR-123 and THR-184 bind the BMP type II receptor BMPR-II and selective BMP type I receptors actR-IA (ALK2) and BMPR-IA (ALK3). Unlike BMP-7, they do not bind BMPR-IB (ALK6).

As explained in Carlson et al. (177), the design of BMP-7 mimetic peptides THR-123 and THR-184 used the x-ray structure of BMP-7 (178) to identify solvent accessible regions likely involved in receptor binding, then identified and optimized the best leads based on *in vitro* assays. These peptides, which are anti-inflammatory, anti-apoptotic and anti-fibrotic, cover the tight beta-turn region of finger 2 (Figure 7A). The fact that they display no osteogenic activity and do not bind to BMPR-IB (ALK6) correlates with the fact that osteogenic peptides identified to date (159–161, 170–172) cover the physically separate “knuckle” region (Figure 7C), and provides a physical explanation for the specificity of activity (Figure 8).

The knuckle region covered by the osteogenic peptides and the beta-turn region covered by the non-osteogenic mimetic peptides described here are separate structural regions (see Figure 7C). This separation allows small mimetic peptides to be designed with specific activity. A cyclized BMP-7 derived peptide, THR-123, located in the beta-turn that is C-terminal to the “knuckle” region (see Figure 7B), was reported to successfully reverse established kidney fibrosis in mouse models of acute and chronic renal injury (149).

THR-123 selectively binds the type I receptors ALK2 and ALK3 but does not bind ALK6. (Figure 8). It exhibits robust anti-inflammatory, anti-apoptotic and antifibrotic and regenerative effects in several experimental models of acute and chronic kidney diseases (149). More recently Li et al. (120) have shown that THR-123, effectively induced nongenetic conversion of human pancreatic exocrine cells to insulin-expressing and functional—glucose-responsive—endocrine cells with a capacity for rapid reversal of diabetes *in vivo* (158). This work independently demonstrates a safer and simpler alternative to genetic reprogramming. Unlike BMP-7, BMP mimetic peptide THR-123 does not induce ectopic bone formation (177).

THR-184, another mimetic peptide of BMP-7 was also designed and optimized. The receptor binding and *in vitro* assay activity—half maximal effective concentrations (EC_50_)—of the lead peptide variants were used for structure/activity analysis to arrive at THR-184 as the prime clinical candidate. Both BMP mimetics (THR-123 and THR-184) selectively bind the BMP type I receptors ActR-IA (ALK2) and BMPR-IA (ALK3) and type II receptor BMPR-II. They do not bind BMPR-IB (ALK6) (Figure 8).

THR-184 was evaluated in clinical studies of Acute Kidney injury (NIH, ClinicalTrials.gov Identifier: NCT01830920) and found to be safe and well tolerated. There was a noticeable reduction in the incidence of AKI in the patient subgroup with pre-existing CKD treated with the highest dose of THR-184 (177). In a type 1 diabetes rat model where islet cells are damaged by Tacrolimus, THR-123 regenerates the islet cells and eliminates the diabetic condition (158). Recently, in mice subjected to transverse aortic constriction (TAC), Salido-Medina et al. (122) established the cardioprotective effects of the two BMP-7 mimetics: THR-123 and THR-184. Daily i.p. injection with either peptide during four weeks, starting on the day of TAC surgery, (i) rescued the expression of BMPR-IA (ALK3) and associated pSMAD1/5/(8)9 signaling in the LV, (ii) prevented transcriptional activation of remodeling-associated genes (Col 1a1, β-MHC, BNP and Acta-2), (iii) attenuated LV structural damage (hypertrophy and fibrosis), and (iv) diminished LV dysfunction (systolic and diastolic). We have shown in a rat left anterior descending artery ligation model that treatment with THR-123 protects cardiomyocytes, and limits infarct size after myocardial ischemic and reperfusion injuries (124). Based on the promise of THR-184, we are currently evaluating its application to rescue DCD hearts from IRI in a rodent model (179). The combination of a perfusate containing THR-184 and normothermic EVHP treatment is a novel and promising donor heart preservation strategy to ameliorate myocardial IRI and to improve cardiac function in the DCD hearts, thereby increasing the number of transplantable grafts in heart transplantation.

## 3 Conclusions & remarks

The supply-demand imbalance of heart allografts available for transplantation continuously grows as more end-stage patients are listed for heart transplantation. Until recently there has been little progress in increasing the supply of hearts (29, 180). The development of Normothermic *ex vivo* machine perfusion (NEVMP), which (despite its limitations), keeps the heart in a near physiological state is a breakthrough that has expanded the availability of donor heart allogeafts. Coupled with other therapeutic modalities to preserve and regenerate the heart, it could lead to new therapies for patients with advanced heart failure and potentially reduce the number of patients in need of a heart transplant.

NEVMP offers protection from ischemia during transportation and allows for evaluation of organs from DCD donors, which are at a higher risk due to the unavoidable warm ischemic conditions involved during determination of donor death. NEVMP allows repeated perfusate sample monitoring of changes in biomarker release, making possible more precise analysis of inflammatory and injury markers such as TNF alpha, IL-6, IL-8 and IL-10 for graft evaluation. Furthermore, this technique permits timely biomarker evaluation to fit within the limited time of heart transplantation protocols. Several biomarkers, such as troponins and lactate, are rapidly and routinely measured in clinical practice and could be implemented without difficulty. The combined use of several biomarker measurements would likely provide a more robust assessment of myocardial injury. Current imaging modalities could also be used to evaluate cardiac grafts, permitting detailed graft characterization. A high-resolution CT scan can detect myocardial fibrosis with good reliability and sensitivity (181). The contrast-enhanced cardiac computed tomography (CCT) was recently demonstrated to be a potentially accurate alternative to Cardiac Magnetic Resonance for the identification of myocardial fibrosis (182). Of particular relevance is a real-time and non-invasive method of assessing graft function and injury based on mitochondrial oxygenation of heart surface tissue using Resonance Raman Spectroscopy (183). This provides a means of predicting the oxygenated state of mitochondrial cytochrome C during perfusion, possibly enabling the assessment of apoptosis. Moreover, *ex vivo* systems present a significant opportunity for the administration of therapeutic interventions to donor hearts. To this end, several new therapeutics have been explored for their efficacy in alleviating cardiac IRI. We designed a series of novel BMP mimetic small peptides which exhibit robust anti-inflammatory, anti-apoptotic and anti-fibrotic and regenerative effects to protect cardiomyocytes and alleviate cardiac IRI *in vivo*. These compounds improve cardiac function and metabolic parameters. Our lead BMP mimetic, THR-184 has been tested clinically and found to be safe and well tolerated by patients in clinical trials. It could quickly enter IND status for the *ex vivo* preservation of DCD hearts.

*Ex vivo* normothermic perfusion combined with new therapeutics such as the BMP mimetics is a novel approach that could overcome the major risk of cardiac ischemic injury and provide more favorable organ utilization and potentially expand the donor pool for heart transplantation. Studies of new therapeutic treatments for the preservation and recovery of donor hearts for transplant will likely serve as a path forward for the development of compounds for the treatment of a broader spectrum of patients with heart failure.

## 4 Significance

Heart Disease is the leading cause of death in the USA. In 2018, there were 6.2 million US adults that had heart failure. In 2022, 702,880 people died from heart disease. In 2019–20 the cost of heart failure/disease to US society was estimated to be $252.2 billion (https://www.cdc.gov/heart-disease/data-research/facts-stats/index.html#cdcreference_3). The only definitive treatment for heart failure is cardiac transplantation.

The development of *ex vivo* heart prefusion technologies has significantly increased the number and quality of donor hearts available for transplantation and offers a potential means for rehabilitating hearts that would otherwise be rejected. Ischemia is an unavoidable effect of current heart procurement protocols, which leads to Ischemia/reperfusion injury to the transplanted heart and other organs of the recipient and continues to limit the potential of this technology.

This paper presents a therapeutic extension of the current and projected near-term techniques used to preserve the heart for transplantation. Success with this therapy could extend the time between explant and implant from hours to days or possibly weeks and dramatically increase the number of donor hearts acceptable for transplantation (currently less than 40%).

The therapeutic we employ is a growth factor mimetic that has been shown to be effective in animal models at protecting and repairing cardiomyocytes damaged by ischemia/reperfusion injury. Repairing this injury means we can treat heart failure and other organs damaged during pre- and post-transplant.

## Data Availability

The original contributions presented in the study are included in the article/Supplementary Material, further inquiries can be directed to the corresponding author.
